# Association between maternal reflective function and preschool children’s cognitive abilities

**DOI:** 10.3389/fpsyg.2022.995426

**Published:** 2022-11-16

**Authors:** Jelena Komanchuk, Deborah Dewey, Gerald F. Giesbrecht, Martha Hart, Lubna Anis, Henry Ntanda, Judy L. Cameron, Nicole Letourneau

**Affiliations:** ^1^Faculty of Nursing, University of Calgary, Calgary, AB, Canada; ^2^Owerko Centre for Children’s Neurodevelopment and Mental Health, University of Calgary Alberta Children’s Hospital Research Institute, Calgary, AB, Canada; ^3^Department of Pediatrics and Community Health Sciences, Hotchkiss Brain Institute, Cumming School of Medicine, University of Calgary, Calgary, AB, Canada; ^4^Department of Pediatrics, Psychiatry, and Community Health Sciences, Cumming School of Medicine, University of Calgary, Calgary, AB, Canada; ^5^Department of Psychiatry, University of Pittsburgh, Pittsburgh, PA, United States

**Keywords:** maternal reflective function, cognitive abilities, attachment security, maternal depression, preschool, regression, APrON study

## Abstract

Children’s cognitive abilities (e.g., working memory) are associated with mental health, adaptive behaviors, and academic achievement, and may be enhanced by parental reflective function (i.e., capacity to reflect on mental states, feelings, thoughts, and intentions in one’s child and oneself). We evaluated associations between maternal reflective function and children’s cognitive abilities alone and while controlling for parent-child attachment and interaction quality, and psychosocial (i.e., maternal depressive symptoms, adverse childhood experiences) and sociodemographic (e.g., socioeconomic status) factors. Our sample, recruited in Canada, was primarily white and included 73 mothers and their 4–5 year old preschool children. Maternal reflective function was measured with the Reflective Functioning Scale applied to the Parent Development Interview and the Parental Reflective Functioning Questionnaire. Multiple regression analyses revealed that maternal reflective function was associated with children’s cognitive abilities. The Parent Development Interview rated child-reflective function was associated with children’s higher verbal comprehension alone and while adjusting for covariates (e.g., parent-child interaction quality, socioeconomic status), and the Parental Reflective Functioning Questionnaire Interest and Curiosity with higher verbal comprehension while adjusting for parent-child interactions and attachment pattern. The Parental Reflective Functioning Questionnaire Certainty in Mental States was associated with higher working memory scores for children while adjusting for covariates. Full Scale IQ and Visual Spatial Index were not significantly associated with maternal reflective function. Associations were found between secure and disorganized attachment with higher verbal comprehension and lower working memory, respectively. These findings highlight the importance of high maternal reflective function to cognitive abilities in early childhood.

## Introduction

A child’s cognitive ability underlies their capacity to process, store, incorporate, and recover information in memory, as well as sustain attention, make decisions, perceive information, learn knowledge, skills, and behaviors, and acquire language such as receptive and expressive skills (Kiely, 2014). Meta-analyses show positive associations between childhood intelligence and adaptive (e.g., communication, daily living, motor skills) behaviors in preschool-aged children (Alexander and Reynolds, 2020), and later academic achievements (Roth et al., 2015). Meta-analytic findings also highlight positive correlations between working memory and educational outcomes, such as reading (Peng et al., 2018) and mathematical performance (Friso-van den Bos et al., 2013). Children with major depressive disorder have demonstrated lower intelligence scores (e.g., full scale, verbal), executive functioning skills (e.g., working memory), and verbal memory compared to their peers (see Wagner et al., 2015 for meta-analytic review). Given associations between children’s cognitive abilities and academic outcomes and mental health, developing a better understanding of early environmental factors that could modify or improve cognitive abilities is necessary to ensure a positive developmental trajectory.

Parental reflective function (RF), is the ability to “mentalize” or envision mental states in oneself and one’s child (e.g., thoughts, feelings, desires, and intentions) and verbalize them (Fonagy et al., 2002, p. 363; Fonagy, 2006; Sharp, 2006; Midgely and Vrouva, 2012). It is a modifiable trait (Byrne et al., 2020; Letourneau et al., 2020; Slade et al., 2020) that is theorized to improve children’s developmental attainment, including cognitive abilities (Steele et al., 1996; Fonagy and Target, 1997; Fonagy et al., 2002; Allen et al., 2008; Sharp and Fonagy, 2008). Interventions designed to improve parental RF have demonstrated improvements in infants’ speech development (Bain, 2014), reductions in school-aged children’s externalizing (e.g., aggressiveness) behaviors (Londono Tobon et al., 2020), and improvements in preschool-aged children’s developmental outcomes (Anis et al., 2020a). Given the importance of children’s cognitive abilities and modifiability of parental RF, further research is needed to examine whether parental RF is associated with preschool children’s cognitive abilities.

### Parental reflective function

RF is the capacity that allows individuals to make sense of one another; parents with high RF have the ability to see their child as an autonomous individual with a mind of their own (Fonagy et al., 1991, 1993). For example, when a child is upset, a highly reflective parent can recognize and accurately address the source of the child’s distress by considering how the child is thinking and feeling. In contrast, a parent with low RF would possibly ignore or misinterpret the source of the child’s distress. The construct of RF is the operationalization of mentalization (i.e., ability to understand others and oneself in terms of mental states) in the context of attachment relationships and is frequently used synonymously with mentalizing (Fonagy et al., 1998; Sharp and Fonagy, 2008). RF is distinguishable from many related terms including “mindblindedness,” “mindreading,” “theory of mind,” “metacognition,” “mindfulness,” “empathy,” and “emotional intelligence” (Fonagy and Target, 1997; Fonagy et al., 2002; Sharp et al., 2006; Allen et al., 2008). The related concept of “mind mindedness,” defined as the “proclivity to treat one’s child as an individual with a mind” (Meins and Fernyhough, 1999, p. 364), is a commonly used construct. While parental RF refers to a parent’s ability to mentalize, mind mindedness focuses on how frequently parents spontaneously mentalize (Medrea and Benga, 2021). Although the constructs are not synonymous, Sharp and Fonagy (2008) suggest that mind mindedness may tap into the “same underlying neurobiological socio-cognitive system” as RF (p. 737).

### Parental reflective function and children’s cognitive abilities

There is a paucity of research investigating the associations between parental RF and children’s verbal comprehension, IQ, and visual spatial skills. Therefore, we broadened our literature review to include research on parental RF and other cognitive abilities, such as executive function, and found some support for an association. A longitudinal path analysis in Canada (*n* = 385) found that maternal cognitive sensitivity (e.g., parental ability to cognitively stimulate their child) mediated the association between maternal RF (referred to as maternal reflective capacity), measured when children were 18 months old, and 4-year-old children’s executive functioning, theory of mind, and receptive language (Wade et al., 2018). Studies have also reported that maternal RF is positively associated with school-aged children’s RF (Ensink et al., 2015; Rosso and Airaldi, 2016), and as RF demands cognitive abilities, such as perceiving and processing mental states and emotions (Slade, 2005; Benbassat, 2020), the results of these studies support a link between maternal RF and children’s cognitive abilities.

Despite limited research on empirical associations between parental RF and children’s cognitive abilities, programs designed to promote parental RF are proliferating, and research is emerging on the effect of parental RF-based programs on children’s cognitive abilities (e.g., Sleed et al., 2013; Bain, 2014; Longhi et al., 2019). Programs designed to foster maternal RF improved infant’s cognitive outcomes in two studies, yet maternal RF was not reported (Sleed et al., 2013) and associations between maternal RF and children’s cognitive outcomes were not evaluated (Bain, 2014). In one study, mother-infant dyads experiencing homelessness were recruited to evaluate a psychotherapeutic baby clinic (Sleed et al., 2013). Infants participated in cognitive assessments using the Bayley Scales of Infant Development, 2nd edition (e.g., memory, verbal communication, sensory/perceptual acuity; Bayley-II; Bayley, 1993). Compared to the control group (*n* = 29), infants in the intervention group (*n* = 30) showed significant improvements in cognitive and motor scores from pre- to post-intervention (Sleed et al., 2013). Similarly, mother-infant dyads were recruited from shelters for families experiencing homelessness in Johannesburg to evaluate the effect of Best Beginnings on infant speech development (Bain, 2014). Evaluation of 16 mother-child dyads in the intervention group and 6 mother-child dyads in the control group revealed that infants in the intervention group attained significantly higher scores on a measure of speech development post-intervention compared to infants in the control group. However, the association between maternal RF and children’s cognitive abilities was not assessed.

In two other studies, RF-based programs did not improve children’s cognitive outcomes (Fonagy et al., 2016b; Longhi et al., 2019), measured with the Bayley-III (Bayley, 2006). First-time, young mothers experiencing high psychosocial risk (e.g., adolescent parent, eligible for income support) participated in a randomized controlled trial to evaluate the effect of Minding the Baby^®^ on child developmental outcomes (Longhi et al., 2019). The intervention commenced prenatally and continued until the children were 2 years of age (Slade et al., 2010, 2020; Sadler et al., 2013), and results revealed no significant difference in children’s cognitive abilities between the intervention and control groups at 2 years (Longhi et al., 2019). In contrast, 76 mother-infant dyads were randomized to receive a parent-infant psychotherapy (PIP) program or treatment-as-usual (Fonagy et al., 2016b). PIP involved at a minimum, bi-weekly sessions that had the mother discuss their own mental state and factors affecting it. At 1 year of age, no statistically significant differences were found between the intervention and control groups with children’s cognitive abilities, maternal RF assessed using the Parent Development Interview (Slade et al., 2004), maternal-child attachment, or maternal-infant interactions. The association between maternal RF and children’s cognitive abilities was also not examined (Fonagy et al., 2016b).

Although correlational and experimental studies have been conducted on maternal RF and children’s cognitive abilities, several gaps exist in the literature. Despite showing some promise of an association, correlational research is lacking on children’s verbal comprehension, visual spatial skills, and Full Scale IQ. Randomized controlled trials have been conducted, which offer numerous benefits, such as minimizing bias and increasing capacity to establish causality (Hariton and Locascio, 2018). However, interventional research on parental RF-based programs and children’s cognitive abilities to date has focused on infants, and has either not measured parental RF or not evaluated the association between parental RF and children’s cognitive abilities. Therefore, additional research, focused on preschool-aged children, is needed to understand the association between maternal RF and children’s cognitive abilities, including Full Scale IQ, working memory, verbal comprehension, and visual spatial skills.

### Reflective function and parent-child interaction quality

Slade (2005) contended that parents with high RF understand how mental states influence their children’s and their own behaviors, thus they can anticipate their child’s actions, and utilize this awareness to guide their caregiving interactions with their children. Indeed, a narrative review of 47 studies concluded that parental RF was positively associated with caregiving qualities, such as maternal sensitivity toward their child (Camoirano, 2017). Caregiving interactions characterized by parental sensitivity at 10–12 months have been associated with higher cognitive scores for children at 18 months on the Bayley-II (Bayley, 1993; Malmberg et al., 2016). Parental sensitivity at 24 months (*N* = 630) was also associated with higher scores on the Wechsler Preschool and Primary Scales of Intelligence (Wechsler, 1974) in children 36 months of age (Mills-Koonce et al., 2015). Therefore, caregiving qualities such as parental sensitivity could confound observed associations between parental RF and preschool children’s cognitive abilities and need to be considered as a covariate in analyses of parental RF and children’s cognitive abilities.

### Parental reflective function, parent-child attachment, and cognitive abilities

An association between parents’ RF and attachment security has also been reported in several studies (e.g., Fonagy et al., 1991; Stacks et al., 2014). For example, Slade et al. (2005a) evaluated maternal RF with Fonagy et al. (1998) RF scoring of the Parent Development Interview (Slade et al., 2004, 2005b) in a sample of 40 mothers with infants. Infants with secure attachment patterns at 14 months had mothers with significantly higher RF scores when the infant was 10 months of age than infants with disorganized or resistant attachment patterns (Slade et al., 2005a). Similarly, maternal RF, measured with the Parent Development Interview-Revised Short Form (Slade et al., 2003, 2005b) and infant attachment were assessed at 16 months in a sample of 83 mothers with histories of maltreatment (Stacks et al., 2014). Maternal RF was positively correlated with secure (vs. avoidant or disorganized) attachment patterns, and parental sensitivity mediated the link between maternal RF and infant attachment security (Stacks et al., 2014). Further, higher parental RF, measured using the Parental Reflective Functioning Questionnaire (Luyten et al., 2017), predicted secure (vs. insecure) infant attachment patterns (Fonagy et al., 2016a). Parent-child attachment patterns have also been associated with executive function (e.g., Bernier et al., 2015; Regueiro et al., 2020), cognitive abilities on the Bayley-II (Bayley, 1993; Ding et al., 2014), IQ on Wechsler’s Intelligence Scale for Children—IV (Wechsler, 2003; Almas et al., 2016), and children’s mentalizing, especially “thinking-about-feeling,” abilities (see Zeegers et al., 2019 for review). Therefore, parent-child attachment is an important covariate to consider when examining the association between parental RF and children’s cognitive abilities.

### Sociodemographic and psychosocial influences on children’s cognitive abilities

There are also significant associations between socioeconomic status (SES), child sex, parental depression, and children’s cognitive abilities. Interrelated factors such as maternal education (Harding, 2015; Harding et al., 2015), income (Dickerson and Popli, 2016; Khanam and Nghiem, 2016), marital status (Son and Peterson, 2017), as well as overall SES can have significant effects on children’s cognitive abilities, including executive functioning, language development, and academic achievement (see Korous et al., 2020 for a systematic review of meta-analyses). Extensive research has linked maternal depression to poorer cognitive abilities in children, such as lower receptive vocabulary (Letourneau et al., 2013; Kingston et al., 2015; Liu et al., 2017). Child sex has also been associated with children’s cognitive abilities; for example, boys have performed lower on a measure of cognitive abilities (Bayley Scales of Infant and Toddler Development and McCarthy Scales of Children’s Abilities; McCarthy, 1972), than girls in the context of maternal depression (see Ahun et al., 2021 for meta-analysis). Consequently, SES, maternal depression, and child sex should be considered as a covariate in analyses of pre-schoolers’ cognitive abilities.

Although significantly less studied, maternal adverse childhood experiences (ACEs), such as abuse and neglect, have been associated with children’s development. Maternal ACEs are associated with smaller intracranial volumes in infants (Moog et al., 2018), increased risk for physical, emotional (Madigan et al., 2017), and behavioral concerns (Letourneau et al., 2019), and suspected developmental delays (Folger et al., 2018). Therefore, maternal ACEs are an important covariate to consider in studies examining the association between maternal RF and children’s cognitive abilities.

The current study addresses the following question: What is the association between maternal RF and preschool children’s cognitive abilities alone and after adjusting for important covariates, such as parent-child interaction, attachment pattern, psychosocial (i.e., depression, ACEs), and sociodemographic (e.g., SES) variables? We predicted that higher parental RF would associate with higher children’s cognitive abilities, alone and accounting for: (1) relationship qualities (i.e., parent-child interaction and attachment) and (2) psychosocial (i.e., depression, maternal ACEs) and sociodemographic (e.g., socioeconomic status) variables.

## Materials and methods

### Study design and sample

This longitudinal correlational study utilized a subsample of parents with preschool children from the Alberta Pregnancy Outcomes and Nutrition (APrON) longitudinal pregnancy cohort. Comprehensive APrON study details have previously been published (Kaplan et al., 2014). In brief, pregnant women, 16 years and older with a gestation less than 27 weeks were recruited from maternity care and ultrasound clinics in Alberta, Canada to participate in the APrON study. Women who could not complete questionnaires in English or who planned to relocate from the study region within 6 months were excluded. In this sub-study, families with children who had intellectual or motor disabilities, or a genetic disorder were excluded. In addition, only families for whom we had parental RF, parent-child interaction quality, parent-child attachment, maternal ACEs, demographic and psychosocial data, and child cognitive outcomes at 4–6 years were included. This resulted in a subsample of 73 mother-child pairs with RF and child cognitive outcome data. Ethics approval was obtained from the University of Calgary Conjoint Health Research Ethics Board (ID: REB15-1200) and mothers provided informed consent for their participation and that of their children.

Data on children’s cognitive abilities were measured with the Wechsler Preschool and Primary Scale of Intelligence–Fourth Edition (WPPSI-IV) when children were on average 53.6 months (*SD* = 5.1) and maternal RF was collected during a clinic visit when children and mothers were, on average, 60.0 (*SD* = 3.82) months and 37.6 (4.23) years, respectively. Maternal data on ethnic identity, education, income, marital status, country of birth, child sex, gestational age, and birth weight was collected at a clinic visit that occurred when the child was 3 months of age or via mail, and maternal ACEs were collected cross-sectionally via email when children were 27.35 months (*SD* = 4.06). Data on maternal-child attachment pattern and interaction quality were collected at 21.09 months (*SD* = 3.47) and 5.61 (*SD* = 0.34 months), respectively.

#### Measures

We measured maternal RF by (1) coding the Parent Development Interview (Slade et al., 2004) with the Reflective Functioning Scale (Fonagy et al., 1998; Slade et al., 2005b) and (2) attaining parental self-reported RF with the Parental Reflective Functioning Questionnaire (PRFQ) (Luyten et al., 2017) to determine if maternal RF measured with the gold standard interview-based tool and newer self-report RF questionnaire both showed associations with children’s cognitive abilities. Observational methods were employed to obtain covariate data on maternal-child interaction quality and attachment patterns. Parental surveys provided covariate data for psychosocial variables (i.e., maternal ACEs and depressive symptoms). Demographic data were also collected via surveys to describe the sample and as covariates. Children’s cognitive abilities were assessed with the WPPSI-IV (Wechsler, 2012).

##### Independent variable

###### Reflective function

We assessed maternal RF with the Reflective Functioning Scale, which was developed by Fonagy et al. (1998), and was later adapted (Slade et al., 2005b) to code semi-structured Parent-Development Interviews (PDI) (Slade et al., 2004). During the 20-item semi-structured PDI interviews, parents describe their own and their child’s behaviors, thoughts, and feelings in different parenting situations (Slade et al., 2004). The interviews were audio-recorded and transcribed verbatim. Trained coders (i.e., author 6 and a master coder/trainer from The New School University, NYC, USA), scored each question using the Reflective Functioning Scale (Fonagy et al., 1998; Slade et al., 2005b) to assess parental insight into their own and their child’s thoughts, feelings, mental states, and intentions as a parent in the parent-child relationship. Scores are derived, each ranging from -1 (anti-reflective; parent attempts to evade question, responds with hostility, or offers bizarre and incoherent answers) to +9 (exceptionally reflective; parent provides sophisticated, complex, coherent answers) to evaluate (1) **self RF,** representing the parent’s ability to recognize their own thoughts, feelings, intentions, and mental states, and (2) **child RF**, demonstrating the parent’s ability to recognize their child’s thoughts, feelings, mental states, and intentions. Two points were added to each score for a range of 1–11, to aid in analysis interpretation. To ensure coders were blind to other participant data, deidentified transcripts were provided. We evaluated inter-rater reliability via percentage agreement between the coders in 15 interviews and observed 86 and 80% agreement for the self and child RF scale scores.

The PRFQ is an 18-item self-reported questionnaire that evaluates parental RF (Luyten et al., 2017). Individuals rate each item on a seven-point Likert scale, ranging from 1 (strongly disagree) to 7 (strongly agree). This measure takes approximately 5 min to complete. We employed the *Certainty in Mental States* scale, which assesses parental ability to recognize and comprehend the opacity of mental states, and the *Interest and Curiosity* scale, which investigates parental interest and curiosity in their child’s mental states. For *Certainty in Mental States* and *Interest and Curiosity*, mid Likert-scale answers indicate high RF. Scores were transformed as per Anis et al. (2020b) method to ensure that lower scores corresponded to lower RF and higher scores indicated higher RF on the 1–7 scale. The PRFQ has demonstrated good internal consistency, with the following reported Cronbach’s alphas: *Certainty in Mental States* = 0.82 and *Interest and Curiosity* = 0.75 (Luyten et al., 2017). Anis et al. (2020b) demonstrated that PRFQ and PDI-Reflective Functioning Scale scores were significantly associated, with original and transformed coding (e.g., *r*’s = 0.22–0.30).

##### Covariates

###### Attachment

Maternal-child attachment security was measured with the Strange Situation Procedure, which is considered the gold-standard measure for attachment in children (Ainsworth et al., 1978; van IJzendoorn et al., 1992), and has been utilized in children up to 40 months of age (Śliwerski et al., 2020). The Strange Situation Procedure was administered in a laboratory setting, and children’s behaviors were assessed throughout eight stressful, yet brief episodes, such as the parent leaving their child in a room with a stranger present. A trained coder (author 6) classified dyads according to four attachment categories, Avoidant (A), Secure (B), Resistant/Ambivalent (C) and Disorganized (D), attaining 100% intra-rater reliability on assessments coded 1 month apart (Ainsworth et al., 1978; Main and Solomon, 1986). Author 6 was trained and certified in ABC coding by Alan Sroufe at the University of Minneapolis (achieving > 80% reliability) as well as by Marinus van IJzendoorn at the University of Cambridge in D coding (achieving > 80% reliability). She also attained 80% inter-rater reliability on ABCD with this SSP dataset with Elizabeth Carlson at the University of Minneapolis. Similar with previous research, individuals were dichotomized as secure vs. insecure and disorganized vs. organized (Cassidy et al., 2011; Letourneau et al., 2015).

###### Parent-child interaction quality

This was measured with the Total Scale score of the observational Parent-Child Interaction Teaching Scale (PCITS) (Oxford and Findlay, 2013). The PCITS is considered the gold standard measure of parent-child interaction quality, which is assessed while a parent teaches their child a novel skill. Seventy three binary items (yes, no) are used to assess parental (e.g., sensitivity to cues, response to distress) and child (e.g., clarity of cues) behaviors. Scores range from 0 to 73, with higher scores indicating higher quality interactions. The PCITS can take up to 5 min to complete and parent-child dyads were recorded to enhance coding accuracy. A certified coder (i.e., attained > 90% reliability with University of Washington standardized coding), blind to other participant data, coded videos for parental sensitivity to cues, responsiveness to distress, social-emotional growth fostering, cognitive growth fostering, and children’s clarity of cues and responsiveness to their parent. Intra-rater reliability was assessed with every 10^th^ video, and 95% intra-rater agreement was maintained.

###### Psychosocial variables

We employed the widely utilized 10-item self-report ACE Questionnaire to determine mothers’ exposure to ACEs from birth to 18 years (Felitti et al., 1998/2019). ACEs include exposure to child maltreatment (i.e., abuse and neglect) and household dysfunction (e.g., domestic violence). Individuals receive a score of one for each type of exposure, and scores can range from 0 to 10. Excellent internal consistency (α = 0.88) has been demonstrated with the ACE Questionnaire (Murphy et al., 2014). Maternal ACEs were classified as 0, 1, 2, or ≥ 3. Maternal depressive symptoms were measured with the Edinburgh Postnatal Depression Scale (EPDS), which is a self-reported 10-item questionnaire that requires parents to recall depressive symptoms during the previous week (Cox et al., 1987). Scores can range from 0 to 30, with higher scores associated with more severe depression. Although the tool is not utilized to diagnose postpartum depression, it is the most widely employed screening tool for postpartum depression (Gibson et al., 2009). Maternal depressive symptoms were measured at 3, 6, 12, 18, 24, and 36 months postpartum, and we utilized mothers’ mean EPDS scores over time to capture mothers’ symptoms in the years prior to the child’s cognitive assessment.

###### Demographic variables

We collected child demographic data on sex, gestational age, and birth weight, and maternal data on education, ethnicity, income, marital status, and country of birth. Composite measures of SES often include educational level, income, and marital status constructs (Letourneau et al., 2011). Similar to other research (e.g., Ali et al., 2020), SES was calculated from maternal income, education, and marital status by summing the values according to the follow dichotomies: income < $70,000 (0) or ≥ $70,000 (1), educational attainment of < university degree (0) or ≥ a university degree (1), and not married (0) or married (1). The sample’s SES scores ranged from 1 to 3, with higher scores indicative of a higher SES.

##### Outcome

###### Children’s cognitive abilities

We measured children’s cognitive abilities with the Wechsler Preschool and Primary Scales of Intelligence–Fourth Edition, Canadian (WPPSI-IV^CND^), a broad measure of cognitive abilities for children aged 2 years 6 months to 7 years 7 months (Wechsler, 2012). For all children, Full Scale IQ and three composite index scores were calculated (i.e., Verbal Comprehension Index, Visual Spatial Index, and Working Memory Index). Scores have a mean of 100 (*SD* = 15; range: 40–160), and higher scores indicate better performance. Excellent internal consistency has been observed for Full Scale IQ (*r* = 0.95), Verbal Comprehension Index (*r* ≥ 0.93), Visual Spatial Index (*r* = 0.88), and Working Memory Index (*r* ≥ 0.91) (Soares and McCrimmon, 2013).

### Statistical analysis

The sample characteristics were described using frequencies, means, and standard deviations, as appropriate. Regression modeling was employed to determine the relation between maternal RF and cognitive abilities (i.e., Full Scale IQ, Verbal Comprehension, Visual Spatial, Working Memory). Three models were run to (1) evaluate direct associations between maternal RF and children’s cognitive abilities and (2) determine if the association held when accounting for important covariates (Pourhoseingholi et al., 2012). *Model 1* examined the associations between maternal RF and preschool children’s cognitive abilities. *Model 2* examined the association between maternal RF and children’s cognitive abilities while controlling for parent-child interaction quality and attachment pattern. *Model 3* examined the association between maternal RF and preschool children’s cognitive abilities while controlling for parent-child interaction quality and attachment patterns, and maternal psychosocial and sociodemographic variables. The variables met assumptions for normality, linearity, homoscedasticity, and independence. Variable selection for inclusion was based on a combination of assessment of theoretical importance and empirical observations from bivariate correlations. Regardless of the significance in bivariate correlations with measures of children’s cognitive abilities, theoretically important covariates for model two included parent-child interaction quality and attachment pattern (i.e., secure vs. insecure, organized vs. disorganized) and for model three included maternal depressive symptoms and ACEs. In contrast, sociodemographic variables for inclusion in model three were identified by covariate correlations of ≥0.20 to ensure evidence of an association and retain statistical power to detect associations (Bursac et al., 2008). Given that we had five measures of RF, to reduce the possibility of multicollinearity, we relied on bivariate correlations to assist with RF variable selection (see Table 2).

**TABLE 1:** Sample demographic and descriptive characteristics.

Variables	Frequency	Percentage
**Sex**		
Male	33	45.21
Female	40	54.79
**Born in Canada**		
No	16	21.92
Yes	57	78.08
**Ethnicity**		
White	64	87.67
Other	9	12.33
**Marital status**		
Single/divorced	4	5.48
Married/common law	69	94.52
**Maternal education**		
No degree	15	20.55
Degree	58	79.45
**Household income**		
Below 70 k	14	19.18
70 k or more	59	80.82
**Attachment patterns**		
Disorganized	7	9.86
Not disorganized	64	90.14
Secure	37	52.11
Insecure	34	47.89
**Maternal depressive symptoms**		
Depressive score ≤ 9	21	28.77
Non-depressive score ≥ 10	52	71.23
Depression	4.34 (mean)	3.12 (SD)
**Maternal ACEs**		
0	37	50.68
1	11	15.07
2	11	15.07
3 or more	14	19.18
**Socioeconomic status (see methods for classification)**		
1	7	9.59
2	19	26.03
3	47	64.38
	**Mean**	**SD**
Birth weight (grams)	3476.37	545.38
Gestational age at birth	39.49	1.43
PCITS total	52.49	7.60
Full scale IQ	104.78	11.26
Verbal comprehension index	107.04	12.91
Visual spatial index	104.35	13.55
Working memory index	103.11	13.05
PRFQ interest and curiosity	6.61	0.43
PRFQ certainty in mental states	6.03	1.09
PDI child RF	5.27	1.24
PDI self RF	5.40	1.46

PCITS, Parent-Child Interaction Teaching Scale; PRFQ, Parental Reflective Functioning Questionnaire; PDI, Parent Development Interview; RF, Reflective Function.

**TABLE 2:** Bivariate Pearson correlations with maternal and child variables (*N* = 73).

	Variables	1	2	3	4	5	6	7	8	9	10	11	12	13	14
1	Full Scale IQ	–													
2	Verbal comprehension	**0.66** ^a^	–												
3	Visual spatial	**0.63** ^a^	**0.27** ^a^	–											
4	Working memory	**0.59** ^a^	0.13	**0.27** ^a^	–										
5	PDI Child RF	0.08	0.23^b^	0.18	–0.06	–									
6	PDI Self RF	0.10	0.15	0.18	0.05	**0.79** ^a^	–								
7	PRFQ Interest and curiosity	0.11	0.21^b^	0.09	0.11	–0.12	–0.11	–							
8	PRFQ Certainty in mental states	0.07	0.15	0.08	0.22^b^	0.08	0.05	0.16	–						
9	PCITS Total	**0.26** ^a^	**0.25** ^a^	0.03	**0.25** ^a^	–0.01	–0.02	0.12	0.15	–					
10	Disorganized attachment	–0.14	0.03	**−0.25** ^a^	**−0.26** ^a^	–0.09	–0.08	–0.07	0.12	–0.16	–				
11	Secure attachment	0.13	0.09	0.18	0.09	–0.003	0.10	0.14	0.08	0.08	**−0.35** ^a^	–			
12	Depression	–0.14	−0.22^b^	–0.09	0.08	0.07	0.07	–0.05	–0.18	–0.08	–0.07	0.09	–		
13	Maternal ACEs	–0.08	–0.13	−0.22^b^	0.03	–0.09	0.06	0.12	–0.09	0.18	0.07	0.04	**0.28** ^a^	–	
14	Born Canada	0.12	0.19	0.08	0.06	**0.38** ^a^	**0.24** ^a^	0.10	–0.03	–0.05	0.18	**−0.31** ^a^	0.14	–0.15	–
15	Socioeconomic status	**0.41** ^a^	**0.30** ^a^	**0.23** ^a^	0.14	0.02	0.07	**0.29** ^a^	0.04	–0.06	–0.13	–0.18	**−0.31** ^a^	–0.12	0.14

Bolded correlations are significant at ^a^*p* < 0.05, except ^b^*p* < 0.10.

PDI, Parent Development Interview; RF, Reflective Function; PRFQ, Parental Reflective Functioning Questionnaire; PCITS, Parent-Child Interaction Teaching Scale; ACEs, Adverse Childhood Experiences.

All sociodemographic variables in the measures section were considered in the bivariate correlations, but only those with significant associations were reported.

Models were tested for multicollinearity with variance inflation factor (VIF) analyses. A VIF of 5–10 or greater indicates the presence of collinearity concerns (Shrestha, 2020); all VIF values were between 1 and 1.5, suggesting absence of multicollinearity. We also conducted an *a priori* power analysis with G*Power Analysis for a fixed linear multiple regression model (Faul et al., 2007). Given alpha = 0.05, power = 0.80, and an effect size of *r* = 0.23 derived from data showing a significant association between maternal RF and receptive language, an exemplar of cognitive abilities (Wade et al., 2018), our sample size of 73 provided the power to include 9 variables in each model. There was no missing data in our sample of 73 individuals. Given the nature of this study and that we included a limited number of specific RF variables selected *a priori* and with directional hypotheses, a Bonferroni correction for multiple comparisons was not applied (Rothman, 1990).

## Results

### Sample characteristics

Demographic and descriptive data are presented in Table 1. Mothers were an average of 37.6 (*SD* = 4.2) years of age at the RF assessment. The majority were born in Canada (78%), married/common law (95%), had attained a minimum of a university degree (79%), and reported ≥ $70,000 yearly household income (81%). Most mothers did not meet the EPDS threshold (i.e., EPDS score ≥ 10) for mild depressive symptoms (71%) and approximately half of the sample (51%) reported no ACEs. On average maternal scores on the PRFQ *Interest and Curiosity* and *Certainty in Mental States* scales were 6.6 and 6.0, respectively. Average PDI child and self RF scores were 5.3 and 5.4, respectively. Child sex was approximately equivalent (55% female). Most children were born full-term (*M* = 39.5 weeks gestation) and were classified with not disorganized (90%) attachment patterns. Approximately half (52%) presented with secure attachment patterns. On average, children had a Full Scale IQ of 104.8. The mean score on the Verbal Comprehension Index was 107, Visual Spatial Index was 104.4, and Working Memory Index was 103.1. Bivariate correlations are presented in Table 2. Of the associations between RF measures and children’s cognitive abilities outcomes, all demonstrated significant associations except for the PDI self RF scale, thus this variable was not included in models.

### Association between maternal reflective function and preschool children’s cognitive abilities

Linear regression models are presented in Tables 3, 4. Model 1 examined the association between maternal RF (i.e., PRFQ scales and PDI-RF scales) and cognitive abilities. No significant associations were found between RF and Full Scale IQ, the Visual Spatial Index score, or the Working Memory Index score. However, significant associations were found between the PDI child RF scale and Verbal Comprehension [*b* = 2.55 (1.21), *p* = 0.039]. Model 1 explained 12% of the variance in Verbal Comprehension.

**TABLE 3:** Verbal comprehension and working memory stepwise linear regression.

Variables	Verbal comprehension index composite			Working memory index		
	Model 1	Model 2	Model 3	Model 1	Model 2	Model 3
Intercept	107.60 (1.48) *p* < 0.001	79.95 (10.56) *p* < 0.001	69.59 (12.23) *p* < 0.001	103.25 (1.58) *p* < 0.001	92.17 (11.59) *p* < 0.001	80.71 (13.64) *p* < 0.001
PRFQ interest and curiosity	6.56 (3.50) *p* = 0.065	**7.25 (3.51) *p* = 0.043**	6.48 (3.61) *p* = 0.078	2.14 (3.77) *p* = 0.571	1.16 (3.84) *p* = 0.763	–0.58 (4.01) *p* = 0.884
PRFQ certainty in mental states	1.07 (1.39) *p* = 0.442	–0.45 (1.43) *p* = 0.754	–0.71 (1.43) *p* = 0.619	2.61 (1.48) *p* = 0.08	**3.32 (1.52) *p* = 0.034**	**3.70 (1.57) *p* = 0.022**
PDI child RF	**2.55 (1.21) *p* = 0.039**	**2.42 (1.21) *p* = 0.047**	**2.42 (1.20**) ***p* = 0.049**	–0.79 (1.29) *p* = 0.543	–0.78 (1.29) *p* = 0.547	–0.84 (1.32) *p* = 0.526
PCITS total	–	**0.47 (0.19) *p* = 0.019**	**0.49 (0.19) *p* = 0.014**	–	0.24 (0.21) *p* = 0.259	0.19 (0.21) *p* = 0.358
Disorganized attachment	–	7.38 (5.19) *p* = 0.161	9.99 (5.22) *p* = 0.061	–	**-11.87 (5.65) *p* = 0.040**	–10.57 (5.82) *p* = 0.075
Secure attachment	–	5.40 (3.16) *p* = 0.09	**6.75 (3.14) *p* = 0.036**	–	–1.53 (3.43) *p* = 0.658	–0.92 (3.50) *p* = 0.793
Depressive (mean value)	–	–	–0.10 (0.57) *p* = 0.862	–	–	0.41 (0.63) *p* = 0.515
Maternal ACEs	–	–	–1.56 (1.30) *p* = 0.235	–	–	0.63 (1.43) *p* = 0.661
Socioeconomic status	–	–	3.95 (2.40) *p* = 0.105	–	–	4.19 (2.64) *p* = 0.117
R-squared	0.12	0.21	0.28	0.06	0.17	0.21

Significant coefficients (*p* < 0.05) are bolded.

PDI, Parent Development Interview; RF, Reflective Function; PRFQ, Parental Reflective Functioning Questionnaire; PCITS, Parent-Child Interaction Teaching Scale; ACEs, Adverse Childhood Experiences.

**TABLE 4:** Full scale IQ and visual spatial stepwise linear regression.

Variables	Full scale IQ			Visual spatial index (VSI)		
	Model 1	Model 2	Model 3	Model 1	Model 2	Model 3
Intercept	104.92 (1.40), *p* < 0.001	82.04 (10.11), *p* < 0.001	65.07 (11.33), *p* < 0.001	104.08 (1.64) *p* < 0.001	102.49 (12.20) *p* < 0.001	92.21 (14.10) *p* < 0.001
PRFQ interest and curiosity	3.07 (3.32), *p* = 0.357	3.03 (3.36), *p* = 0.371	0.89 (3.35), *p* = 0.792	3.46 (3.89) *p* = 0.378	3.91 (4.07) *p* = 0.341	3.63 (4.30) *p* = 0.391
PRFQ certainty in mental states	0.41 (1.31), *p* = 0.758	–0.53 (1.36), *p* = 0.698	–0.42 (1.33), *p* = 0.751	0.53 (1.54) *p* = 0.729	0.45 (1.64) *p* = 0.783	0.06 (1.65) *p* = 0.973
PDI child RF	0.82 (1.15), *p* = 0.481	0.47 (1.14), *p* = 0.680	0.46 (1.12), *p* = 0.684	2.14 (1.34) *p* = 0.115	1.82 (1.38) *p* = 0.192	1.67 (1.39) *p* = 0.235
PCITS total	–	**0.41 (0.18), *p* = 0.031**	**0.40 (0.17), *p* = 0.031**	–	0.01 (0.22) *p* = 0.958	0.05 (0.22) *p* = 0.816
Disorganized attachment	–	–1.71 (4.98), *p* = 0.732	1.25 (4.84), *p* = 0.796	–	–8.26 (6.03) *p* = 0.176	–5.28 (6.09) *p* = 0.389
Secure attachment	–	3.34 (3.03), *p* = 0.274	4.73 (2.92), *p* = 0.110	–	3.56 (3.64) *p* = 0.331	5.05 (3.64) *p* = 0.170
Depressive (mean value)	–	–	0.11 (0.53), *p* = 0.842	–	–	0.10 (0.66) *p* = 0.875
Maternal ACEs	–	–	–0.64 (1.21), *p* = 0.598	–	–	–2.36 (1.51) *p* = 0.124
Socioeconomic status	–	–	**6.52 (2.23), *p* = 0.005**	–	–	3.51 (2.77) *p* = 0.211
R-squared	0.02	0.13	0.26	0.05	0.11	0.19

Significant coefficients (*p* < 0.05) are bolded.

PDI, Parent Development Interview; RF, Reflective Function; PRFQ, Parental Reflective Functioning Questionnaire; PCITS, Parent-Child Interaction Teaching Scale; ACEs, Adverse Childhood Experiences.

Model 2 adjusted for parent-child interaction quality and attachment pattern. No associations were found between maternal RF (i.e., PRFQ scales and PDI-RF scales) and Full Scale IQ or Visual Spatial Index scores. The PDI child RF [*b* = 2.42 (1.21), *p* = 0.047] and PRFQ Interest and Curiosity [*b* = 7.25 (3.51), *p* = 0.043] scores were associated with Verbal Comprehension. PRFQ Certainty in Mental States [*b* = 3.32 (1.52), *p* = 0.034] was associated with Working Memory. Parent-child interaction quality was positively associated with Verbal Comprehension [*b* = 0.47 (0.19), *p* = 0.019] and Full Scale IQ [*b* = 0.41 (0.18), *p* = 0.031], and parent-child disorganized attachment patterns were negatively associated with Working Memory [*b* = -11.87 (5.65), *p* = 0.04]. Model 2 explained 21% of the variance in Verbal Comprehension and 17% in Working Memory.

Model 3 additionally adjusted for maternal depressive symptoms, ACEs, and SES. No associations were found between the PRFQ or PDI-RF scales and Full Scale IQ or the Visual Spatial Index scores. The associations between PDI child RF and Verbal Comprehension [*b* = 2.42 (1.20), *p* = 0.049], and PRFQ Certainty in Mental States [*b* = 3.70 (1.57), *p* = 0.022] with Working Memory remained. Parent-child interaction quality remained significantly associated with Verbal Comprehension [*b* = 0.49 (0.19), *p* = 0.014] and Full Scale IQ [*b* = 0.40 (0.17), *p* = 0.031]. Associations were observed between parent-child secure attachment and Verbal Comprehension [*b* = 6.75 (3.14), *p* = 0.036], and socioeconomic status with Full Scale IQ [*b* = 6.52 (2.23), *p* = 0.005]. The present model accounted for 28% of the variance in Verbal Comprehension and 21% in Working Memory scores.

## Discussion

The purpose of our study was to determine the association between maternal RF and preschool children’s cognitive abilities, alone and while controlling for parent-child attachment and interaction quality, and relevant psychosocial and sociodemographic variables, such as SES and maternal depression. In model one, we evaluated the association between maternal RF and children’s cognitive abilities. Maternal RF, specifically PDI child RF, that is the mother’s ability to recognize their child’s thoughts, feelings, mental states, and intentions, was positive associated with children’s verbal comprehension. In model two, we assessed the association between maternal RF and children’s cognitive abilities while controlling for parent-child interaction quality and attachment pattern; PDI child RF remained positively associated, and PRFQ Interest and Curiosity showed a positive association, with children’s verbal comprehension. PRFQ Certainty in Mental States was positively associated with children’s working memory. Findings also showed associations between maternal-child interaction quality and children’s verbal comprehension. While controlling for maternal-child interaction quality and attachment, maternal depressive symptoms and ACEs, and SES in model three, PDI child RF and PRFQ Certainty in Mental States remained positively associated with children’s verbal comprehension and working memory, respectively. In the third model, maternal-child interaction quality was positively associated with verbal comprehension, and also associated with Full Scale IQ. No measures of maternal RF were significantly associated with children’s Full Scale IQ or visual spatial skills in any of the models. Overall, our hypothesis that maternal RF would associate with children’s cognitive abilities alone and while adjusting for relevant covariates was supported for verbal comprehension skills and partially supportive for children’s working memory.

Our prospective cohort findings demonstrate a positive association between maternal RF and preschool children’s cognitive abilities, specifically verbal comprehension and working memory. Previous research has shown links between maternal RF and childhood constructs that require complex cognitive abilities, such as school-aged children’s mentalizing (i.e., RF) capacity (e.g., Rosso et al., 2015; Scopesi et al., 2015). Additionally, maternal mind-mindedness (i.e., identification and treatment of their child as an individual with their own mind), a related construct to RF, has been associated with preschool children’s executive function, specifically, inhibitory control (Cheng et al., 2018), child language skills at age two (Bernier et al., 2017), and theory of mind at 5 and 6 years (Kirk et al., 2015). Our findings support the important link between maternal RF and preschool-aged children’s cognitive abilities, specifically, verbal comprehension and working memory. Parental RF has been shown to be a modifiable construct (e.g., Letourneau et al., 2020; Slade et al., 2020); therefore, parental RF interventions have potential to promote children’s cognitive abilities, yet additional research is needed.

The results of this study also demonstrate that RF, whether assessed via a coding scheme applied to a comprehensive interview or parental self-report, is associated with children’s cognitive abilities. This is important for future research and clinical evaluations of parental RF as the PRFQ is a short and accessible questionnaire that can be used to measure parental RF; it takes approximately 5 min to complete, is freely available, requires no specific training, and has potential for widespread use. In contrast, although the PDI-RF is well validated, it is time intensive and requires training.

However, we found differences in associations between the two measures of RF and children’s cognitive abilities. The PDI child RF, which measures parental ability to recognize feelings, thoughts, and intentions underlying their child’s behavior, was associated with verbal comprehension alone and while adjusting for parent-child relationship qualities and psychosocial and demographic variables. Research has shown positive associations between children’s language development and parental contingency (Topping et al., 2013), and parents with high child RF are likely attuned with their child’s readiness (e.g., engagement, disengagement) for interactions. However, maternal PDI child RF remained significantly associated while controlling parent-child interaction quality, which suggests that other aspects of maternal RF encourage children’s verbal comprehension. Research has also shown that exposure to more adult words is associated with children’s higher verbal comprehension (e.g., Huttenlocher et al., 1991; Gilkerson et al., 2018). It is possible that parents who receive high PDI child RF scores also engage in more verbalizations with their child than other parents, especially related to their child’s feelings, thoughts, and mental states. In contrast, the PRFQ Certainty in Mental States was correlated with working memory while adjusting for parent-child relationship qualities and psychosocial and demographic variables. Optimal PRFQ Certainty in Mental States scoring is characterized by moderate certainty (over certainty is seen as intrusive) in their child’s mental states (Luyten et al., 2017). Research has shown that optimal parenting (e.g., low intrusiveness) predicts working memory at 36 months (Wu and Schutte, 2021), which may explain the relation between PRFQ Certainty in Mental States and working memory. This association may also be explained with children’s stress reactivity since preschool children with high cortisol levels have shown lower executive functioning (Blair et al., 2011) and studies have reported associations between low parental RF and children’s emotional regulation abilities (Camoirano, 2017). More research is needed to understand how the PDI-RF and PRFQ differ in relation to children’s cognitive abilities.

Research using Fonagy et al. (1998) Reflective Functioning Scale coding of the PDI (Slade et al., 2004, 2005b) has shown differing associations between parental self RF and child RF. For example, Borelli et al. (2016) found that only child RF was related to child attachment security. In contrast, Suchman et al. (2010) found that self RF was positively correlated with caregiving behaviors (i.e., sensitivity to their child’s cues, social-emotional growth fostering, cognitive growth fostering) in a sample of mothers with drug use disorders. In our analysis, PDI self RF was not significantly associated with children’s cognitive abilities; therefore, we excluded this variable from the regression analysis. Additional research using RF coding of the PDI is needed to understand the associations between parental self and child RF on caregiving behaviors and children’s development.

Although not a central finding, our study also imparts additional knowledge on the association between attachment and children’s cognitive abilities. Disorganized attachment was associated with a noteworthy decrease in working memory by 12 points on a scale that ranges from 40 to 160, which is close to one standard deviation (*SD* = 15) in normative samples (Wechsler, 2012) and nearly one standard deviation (13.05) in working memory scores in our sample; given that a change in scores by half of a standard deviation is frequently considered a clinically significant finding in interventional research (Polit, 2017), our results highlight the clinical relevance of disorganized attachment on children’s working memory. This finding is consistent with Thorell et al. (2012), who reported that disorganized, but not insecure, attachment patterns in school-aged children were significantly related to children’s executive functioning skills, which include working memory. Further, secure attachment was associated with verbal comprehension scores that were nearly 7 points (on a scale with a range of 120 points) higher, when sociodemographic and psychosocial variables were controlled for; secure attachments were not associated with other cognitive abilities, such as visual spatial skills or working memory. Our findings are also consistent with van IJzendoorn et al. (1995) meta-analysis, which reported that insecure attachment patterns were weakly related to lower cognitive (*d* = 0.17) performance and moderately related to language performance (*d* = 0.59). Although other research has shown that RF-centered programs can improve parent-child attachment patterns (Slade et al., 2020), we did not find associations between parental RF and parent-child attachment. However, our samples differed demographically, and we observed a smaller proportion of children with disorganized attachment patterns than Slade et al. (2020) sample. Further research is needed to understand if RF focused interventions support children’s attachment and cognitive abilities.

Previous research has reported associations between sociodemographic variables and cognitive abilities (e.g., Letourneau et al., 2011; Merz et al., 2019). We found that SES was related to Full Scale IQ, which is consistent with extensive literature demonstrating a positive association between SES and cognitive abilities (e.g., Letourneau et al., 2011; Merz et al., 2019). Surprisingly, we did not find significant negative associations between maternal depression and cognitive abilities, despite an abundance of literature showing poorer cognitive abilities among children of mothers experiencing depression (e.g., Liu et al., 2017). However, our sample included very few mothers (29%) with EPDS scores indicative of possible depression. Although no significant relation was found between maternal ACEs and preschool children’s cognitive abilities, this could be due to a modest sample with only 51% of mothers reporting ACEs. Given emerging associations between maternal ACEs and children’s health and development (e.g., Madigan et al., 2017; Folger et al., 2018), the link between ACEs and cognitive abilities should be evaluated with larger and more diverse samples.

## Strengths and limitations

The APrON longitudinal cohort provided an opportunity for prospective analyses (including important covariates) using reliable and valid measures, many of which were observational (i.e., Strange Situation Procedure to measure parent-child attachment patterns, PCITS to assess parent-child interaction quality) as opposed to parent report. Observational assessments of parent-child interaction quality and attachment are considered gold standard measures and we attained high intra-rater reliability for both tools. Administration of the WPPSI-IV provided a rigorous and broad assessment of preschool-aged children’s cognitive abilities. We also employed multiple valid and reliable measures of parental RF. Although other research has shown correlations between the PDI and PRFQ (Anis et al., 2020b), we did not observe these findings, which is a limitation in our research. Further, to our knowledge, an evaluation of association between maternal RF and a broad range of preschool aged children’s cognitive abilities have only been reported by Wade et al. (2018), and their cognitive ability outcomes (i.e., theory of mind, executive function, receptive language) differed from our study. To minimize participant burden, we collected maternal RF and cognitive ability data at differing clinic visits. However, the measurement of children’s cognitive abilities before assessments of parental RF in our study and the correlational analysis pose challenges to inferences about the direction of associations. Given the measurement timing and correlational analysis, our results do not establish causality, and additional research is needed to confirm our findings. Our study included a relatively homogenous, modest sample of low-risk families (i.e., high SES, low ACEs, low depressive scores, primarily Caucasian, married/cohabiting, few young mothers), and it is possible that associations could be stronger in higher-risk populations. Our sample also lacked ethnic diversity and most mothers were born in Canada, which limits generalizability. Further, as the research included only mothers, future research needs to determine if these findings generalize to fathers.

## Conclusion

In conclusion, this study suggests that maternal RF is associated with verbal comprehension and working memory in preschool aged children. Our research also demonstrated the importance of children’s secure and disorganized attachment patterns on verbal comprehension and working memory, respectively. Given the importance of cognitive abilities to children’s mental health, education, and behavioral outcomes, and the modifiability of parental RF and parent-child attachment patterns, these findings underscore the importance of developing early parenting supports to optimize parental RF and parent-child attachment and evaluating the effects of these programs on children’s cognitive abilities.

## Data availability statement

The raw data supporting the conclusions of this article will be made available by the authors, without undue reservation.

## Ethics statement

The studies involving human participants were reviewed and approved by University of Calgary Conjoint Health Research Ethics Board (ID: REB15-1200). The patients/participants provided their written informed consent to participate in this study.

## Author contributions

JK assisted with formulating the research question and data analysis, interpreted results, and wrote the original draft. DD and GFG attained funding for the study, oversaw data collection and coding of select variables, contributed substantive knowledge and methodological expertise, and edited final manuscript. MH collected and coded data and edited final manuscript. LA collected data and edited final manuscript. HN analyzed data and edited final manuscript. JLC provided supervision of JK and edited manuscript. As senior author, NL formulated the research question, attained funding for the study as Principal Investigator, and oversaw data collection, coding, cleaning and analysis, ethics approval and writing of manuscript.
